# Clinical activity and safety of Pembrolizumab in Ipilimumab pre-treated patients with uveal melanoma

**DOI:** 10.1080/2162402X.2016.1143997

**Published:** 2016-02-18

**Authors:** Ioannis Karydis, Pui Ying Chan, Matthew Wheater, Edurne Arriola, Peter W. Szlosarek, Christian H. Ottensmeier

**Affiliations:** a Cancer Sciences Academic Unit, University of Southampton, Southampton, United Kingdom; b Department of Medical Oncology, St Bartholomew's Hospital, London; c Medical Oncology, University Hospital Southampton, Southampton, United Kingdom; d Barts Cancer Institute, Queen Mary University of London, London

**Keywords:** Anti-PD-1, immuno-oncology, metastases, Pembrolizumab, uveal melanoma

## Abstract

**Background**: Untreated metastatic uveal melanoma (UM) carries a grave prognosis. Unlike cutaneous melanoma (CM), there are no established treatments known to significantly improve outcomes for a meaningful proportion of patients. Inhibition of the PD1–PDL1 axis has shown promise in the management of CM and we here report a two center experience of UM patients receiving pembrolizumab. **Methods**: To assess the efficacy and safety of pembrolizumab, we retrospectively analyzed outcome data of 25 consecutive UM patients participating in the MK3475 expanded access program (EAP) who received pembrolizumab at 2 mg/kg 3 weekly. Tumor assessment was evaluated using RECIST 1.1 and immune-related Response Criteria (irRC) by CT scanning. Toxicity was recorded utilizing Common Terminology Criteria for Adverse Events (“CTCAE”) v4.03. **Results**: Twenty-five patients were identified receiving a median of six cycles of treatment. Two patients achieved a partial response and six patients stable disease. After a median follow-up of 225 d median progression free survival (PFS) was 91 d and overall survival (OS) was not reached. There was a significant trend for improved outcomes in patients with extrahepatic disease progression as opposed to liver only progression at the outset. Five patients experienced grade 3 or 4 adverse events (AEs); there were no treatment related deaths. **Conclusions**: Pembrolizumab 2mg/kg q3w is a safe option in UM patients. Disease control rates, particularly in the subgroup of patients without progressive liver disease at the outset are promising; these results merit further investigation in clinical trials possibly incorporating liver targeted treatment modalities.

## Abbreviations

AE: Adverse Event
ALT: Alanine aminotransferase
AST: Aspartate aminotransferase
CM: Cutaneous Melanoma
CTCAE: Common Terminology Criteria for Adverse Events
EAP: Expanded Access Program
ECOG: Eastern Cooperative Oncology Group
irAE: immune-related AE
irRC: immune-related Response Criteria
LDH: Lactate Dehydrogenase
OS: Overall Survival
PD: Progressive disease
PFS: Progression Free Survival
PS: Performance Status
RFA: Radiofrequency ablation
SIRT: Selective internal radiation therapy
TACE: Transcatheter arterial chemoembolization
TIL: Tumor infiltrating lymphocytes
ULN: Upper Limit of Normal
UM: Uveal Melanoma

## Introduction

UM is the most common malignancy of the eye; it is significantly rarer and carries a genetic profile that is markedly discordant from CM. ^1^ UMs typically demonstrate a relatively lower degree of aneuploidy and genomic instability compared with other cancer types ^2-4^ and a mutational load significantly lower than CM. ^5,6^

Even in the pre-immunotherapy and targeted therapy era, outcomes of metastatic UM were worse than metastatic CM with median survival in unselected series as low as 4 mo. ^7^ This possibly relates to the predilection of UM for hepatic metastases—seen in >85% of cases with liver the sole site of metastatic disease in around 50%,^7^ compared to 25% in CM. ^8^

Cytotoxic chemotherapy offers little benefit in UM and is no more effective than in CM when site and extent of disease is considered. ^9^ The absence of activating BRAF mutations in the majority of UM patients limits the use of BRAF inhibitors. Alternative targeted approaches are sought, e.g. the MEK inhibitor selumetinib,^10^ however, at the time of writing none have demonstrated significant activity in large Phase III trials.

Immunotherapy has revolutionized the treatment of metastatic CM; immune checkpoint inhibition with ipilimumab ^11^—an anti-CTLA4 antibody—and anti-PD1 agents alone ^12^ or in combination ^13,14^ results in durable disease control in a significant proportion of patients. UM patients were excluded from taking part in Phase III trials, so evidence of efficacy in this setting is limited.

Small studies and retrospective analyses ^15-17^ have shown that partial and even complete responses to ipilimumab in UM are possible, but rare. Median OS of 5.2–9.6 mo, 1 y OS rates of 22–34% and 1 y PFS rates of <11% were comparable to standard chemotherapy ^10^ but lower than in unselected CM cohorts.

One explanation why immunotherapeutics might be less effective in UM compared to CM could be different driving mutations. ^1,5,18^ Furthermore, the overall mutational load is significantly lower ^5^ compared to CM ^3^ and expression of cancer-testis antigens is significantly rarer. ^19^ Consequently, the number and quality of (neo-)antigens presented to the immune system is likely to be different. Additionally, UM may rely on different immune escape mechanisms most clearly evidenced by the observation that higher numbers of infiltrating T-cells in the primary are linked to a worse outcome. ^20^

Very little information exists in the public domain regarding the efficacy of anti-PD1 agents in UM; the largest case series reported consists of data from seven patients who received pembrolizumab. ^21^ We herein report a two center experience of 25 patients who have received pembrolizumab in in the UK EAP.

## Patients and treatments

### Patient eligibility

Patients treated in the pembrolizumab EAP in our institutions with a diagnosis of metastatic UM were included in this retrospective study. All subjects had received previous ipilimumab and a BRAF inhibitor if eligible. Resolution of AEs due to previous cancer therapy to grade 0 or 1 was required. Additional previous immunotherapies were allowed as long as no severe or life threatening immune-related AEs (“irAEs”) were experienced and there was no ongoing requirement for systemic steroids for the management of irAEs.

Eastern Cooperative Oncology Group performance status (PS) 0/1 was mandated as well as a minimum age of 12. Inclusion criteria included AST and ALT ≤2.5 × upper limit of normal (ULN) or ≤5 × ULN with liver metastases, serum total bilirubin ≤1.5 × ULN or direct bilirubin ≤ULN for patients with total bilirubin level >1.5 ULN. Patients with a history of clinically severe autoimmune diseases, pneumonitis, organ transplant, HIV or active Hepatitis B or C infections and active central nervous system metastases were also excluded. Concomitant systemic antineoplastic therapies were not allowed.

### Treatment

Pembrolizumab was administered at 2 mg/kg in 3-weekly intervals until progression by irRC ^22^, complete response, unacceptable toxicity or for up to 2 y. Frequency of radiological tumor assessment was as per standard of care, typically with body CT scans every 2–3 mo and liver MRIs to optimally monitor liver disease. Blood samples were taken before each infusion to allow assessment of renal, liver, thyroid and bone marrow function for safety and toxicity monitoring. AEs were scored using CTCAE version 4.03.

### Response evaluation

Tumor response was evaluated using the following radiological scoring systems: RECIST 1.1^23^ and irRC.^22^ Best overall response was determined based on irRC criteria where possible to capture delayed antitumor responses.

### Data capture and analysis

Patients receiving pembrolizumab were identified from the oncology pharmacy database. Data was collected retrospectively from patients' notes and electronic records and stored into a Microsoft Access database; statistical analysis and graphing was done using GraphPad Prism Version 6.01. Survival curves were calculated using the Kaplan–Meier method. The log-rank test was used to compare curves and determine the *p* value.

## Results

### Patient characteristics

Twenty-five patients with metastatic UM were enrolled into the pembrolizumab EAP between the 1/06/2014 and 1/8/2015 at our centers. All patients had systemic disease spread at baseline and had received a median of one previous lines of systemic treatment; 11 patients (44%) had also received a median of two liver directed therapies. All patients had previously completed a course of ipilimumab, none had experienced an objective response though nine patients (36%) had a period of stable disease; baseline patient characteristics are presented in Table 1. All seven patients with available cytogenetic results had chromosome three losses and chromosome eight gains in the primary tumors.

**Table 1.** Baseline patient characteristics.

Number of patients		N =	(%)		
a. Demographic Characteristics					
Patient gender	Male	13	52%		
	Female	12	48%		
Eastern Co-operative Oncology Group performance status	0	12	48%		
	1	12	48%		
	2	1	4%		
			Median	Range	
Age at 1^st^ pembrolizumab cycle (y)		58	32–83	
Time from primary diagnosis (mo)		47	9–186	
Time from original systemic recurrence (mo)		11.3	3.7–65.1	
b. Disease Characteristics				
		No. of patients	(%)	
Site of metastatic disease at baseline	Liver only	5	20%	
	Extrahepatic only	6	24%	
	Liver & Extrahepatic	17	68%	
Site of radiological disease progression at baseline	Liver only	11	44%	
	Extrahepatic only	5	20%	
	Liver & Extrahepatic	7	28%	
	None	2	8%	
LDH at baseline	<=ULN	7	28%	
	1-2*ULN	11	44%	
	>2*ULN	4	16%	
Liver function test (ALT/AST and/or bilirubin) abnormalities at baseline	<=ULN	16	64%	
	Grade 1	5	20%	
	Grade 2	3	12%	
c. Previous treatments				
		No. of patients treated (%)		
Liver directed therapy				
	Any	11 (44%)		
	Surgery	3 (12%)		
	Melphalan chemoperfusion	8 (32%)		
	SIRT	2 (8%)		
	TACE	3 (12%)		
	RFA	1 (4%)		
Systemic treatment other than ipilimumab		7 (28%)		
	Interferon α2b	4 (16%)		
	Autologous TILs	1 (4%)		
	Temozolamide	3 (12%)		
	Lomustine	2 (8%)		
	Carboplatin based	2 (8%)		
Previous ipilimumab		25(100%)		
Best response to ipilimumab	Progressive disease	16(64%)		
	Stable disease	9(36%)		

## Response analysis

**Figure 1.**
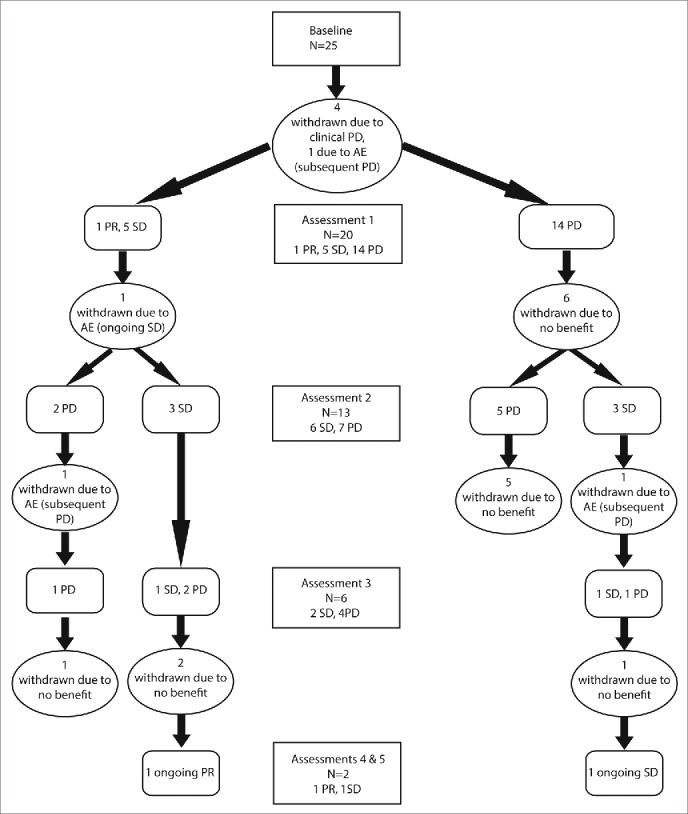
Disease status of UM patients participating in the Pembrolizumab EAP. Treatment was administered at 3 weekly intervals with tumor assessments performed at baseline and every three cycles of treatment or as clinically indicated.

All patients received at least one cycle of pembrolizumab. At data collection cut-off time a median of six cycles of pembrolizumab had been administered per patient and two patients were continuing on treatment. Radiological assessments took place as clinically indicated, typically every three to four cycles (9–12 weeks). Fig. 1 shows a flow diagram outlining treatment course and outcomes.

Four subjects deteriorated rapidly due to disease progression and were withdrawn before the first radiological assessment after receiving only one cycle. Eight patients were withdrawn after having only a single radiological assessment demonstrating disease progression due to rapid clinical deterioration. One patient continuing after an initial scan revealing PD exhibited subsequent disease stabilization and remains on treatment. There were two partial responses, one early that was not maintained and one late that remains on treatment. Table 2 summarizes response types and duration.

**Table 2.** Best radiological disease response to pembrolizumab; nine patients were alive, one with an ongoing partial response and two with stable disease at the time of writing hence median overall and progression free survival was not reached (NR) for several subgroups.

Table 2: Disease Response						
Response	No. of patients	%	Median PFS (d)	Range (d)	Median OS (d)	Range(d)
PR	2	8%	NR (>325)	153–498+	NR (>427)	498–357
SD	6	24%	NR (>293.5)	>112–321+	NR (>405)	286–483
on initial assessment	3	12%	252	>112–321+	NR (>384)	286–483
after initial PD	3	12%	303	>129–293+	NR (>427)	303–431
PD	17	68%	63	7–146	NR (>=163)	7–423
Overall	25	100%	91	9–321+	NR (>=225)	7–498

### Survival analysis

**Figure 2.**
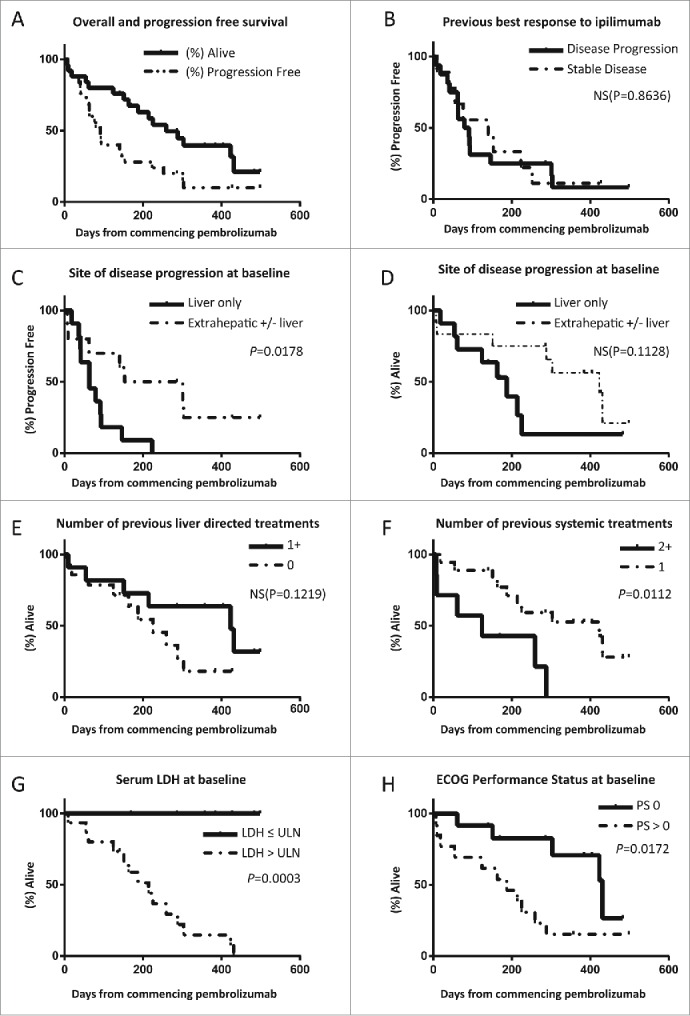
Kaplan–Meier plots of overall and progression free survival of UM patients treated with pembrolizumab at 2mg/kg as part of the expanded access program. (A) Curves for entire group, median OS not reached. (B–H) Curves stratified by best previous response to ipilimumab (B), site of disease progression at baseline (C–D), number of liver directed treatments received prior to enrolling to the EAP (E), number of previous systemic treatments (F), serum LDH (G) and ECOG PS (H).

After a median follow-up of 225 d at the time of data cut-off median, OS was not reached but will be >225 d with a 1 y survival rate of >28%; median PFS was 91 d (Table 2). Eight evaluable patients (32%) achieved disease control (partial response or stable disease) for more than 3 mo and median PFS for this subgroup is projected to be >9.8 mo with a median OS >13.5 mo. A Kaplan–Meier plot of OS and PFS of all patients is presented in Fig. 2A, including censored data for patients who are still responding to treatment.

**Figure 3.**
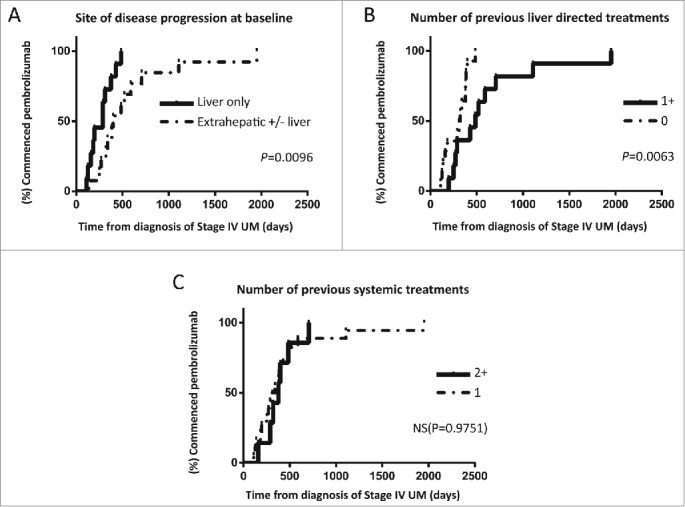
Plots demonstrating lead- in times from diagnosis of UM recurrence to commencing pembrolizumab stratified by (A) location of disease progression at time of commencing pembrolizumab, (B) number of liver directed treatments received prior to enrolling to the EAP and (C) number of previous systemic treatments.

Patients with liver only disease progression at baseline imaging had significantly worse PFS (Fig. 2C) and on immature data there was a trend for worse OS (Fig. 2D). This group had also shorter lead in times from diagnosis of stage IV disease (Fig. 3A), as did patients who had no previous liver directed treatments (Fig. 3B) but in the latter case there was no significant difference in PFS (Fig. 2E).

In contrast, PFS and OS was significantly worse in patients who had more than one previous systemic treatment as compared to only one despite similar lead in times (Figs. 2F and 3C). Previous disease stabilization in response to ipilimumab did not appear to predict response to pembrolizumab (Fig. 2B).

In addition both serum lactate dehydrogenase (LDH) and PS at baseline were associated with a worse PFS and OS (Fig. 2G–H); notably all patients with normal LDH at baseline are still alive as opposed to only one with raised LDH.

## Safety analysis

Pembrolizumab was tolerated well overall with a frequency and type of AEs commensurate with those reported in larger studies.^12^ Five patients (20%) experienced at least one grade 3/4 treatment related AE (Table 3): one patient experienced grade 4 transaminitis after the first dose and one grade 3 skin rash and pruritus after the second dose; both had to discontinue treatment; two patients experienced grade 3 hypophysitis requiring long term steroid replacement, one of these also had an episode of grade 3 diarrhea that settled spontaneously. Finally, one patient experienced grade 3 fatigue and elected to discontinue treatment.

**Table 3.** Treatment related adverse events.

	Any Grade		Grade 3–4	
AE	No. of patients	%	No. of patients	%
Fatigue	8	32%	1	4%
Rash	6	24%	1	4%
Pruritus	5	20%	1	4%
Diarrhea	4	16%	1	4%
Hypophysitis	2	12%	2	8%
Transaminitis	1	4%	1	4%
Pancreatic insufficiency	1	4%	0	
Muscle weakness	1	4%	0	
Oral mucositis	1	4%	0	
Low Testosterone	1	4%	0	
Sjogren's	1	6%	0	

## Discussion

Anti-PD-1 agents are now approved for use in US and European markets for the treatment of metastatic CM pre and post ipilimumab. As entry criteria of the registration trials excluded UM, data on efficacy of anti-PD-1 based immunotherapy in UM is limited.

In our patients, objective response rates were lower than in CM studies,^12^ however, PFS rates were comparable and a significant number (>32%) of patients experienced prolonged (>3 mo) periods of disease stabilization with 28% maintaining disease control for >6 mo.

High LDH and poorer PS at baseline—known markers of disease burden and/or aggressiveness—predicted a shorter duration of benefit. While not surprising this provides additional prognostic information. AEs seen were in line with safety analyses from larger studies ^12^ and confirm that pembrolizumab is well tolerated in this patient population.

The most dramatic observation relates to the role of uncontrolled intrahepatic metastases. Patients with liver deposits as the only site of progression had significantly shorter PFS as compared to subjects with extrahepatic sites (median of 63 vs 153 d, Fig. 2C); additionally, the former uniformly went on to develop disease progression in the liver alone.

The reasons behind this behavior are unclear and likely multifactorial. The liver microenvironment is known to facilitate immune escape^24^ and the specific mechanisms involved may account both for the predilection of UM for liver metastases and the reduced efficacy of immunotherapeutic agents in patients with liver disease. A related and not mutually exclusive possibility is that extrahepatic sites of disease allow better priming of an anticancer immune response which can then control intrahepatic disease. Finally, the pattern of metastatic spread may reflect underlying biological differences—e.g., mutational load, underlying immune escape mechanisms—that influence the ability of checkpoint inhibitors such as pembrolizumab to drive an effective immune response.

While this is a small study, it has significant implications for the management of UM if the findings are confirmed. First, while pembrolizumab may not reproduce the impressive response rates seen in CM, it nevertheless appears to achieve disease control of clinically meaningful duration for a significant proportion of patients, justifying its use in the single agent setting, particularly in the absence of alternative effective systemic treatment options.

Second, active liver metastases in the absence of extrahepatic disease appear to have major prognostic significance as in those circumstances pembrolizumab appears to be ineffective. This finding—if confirmed—advocates against pembrolizumab single agent use in this setting.

Finally, a multimodality approach could target intrahepatic immune escape, utilizing liver directed treatments both prior to commencing pembrolizumab and/or during treatment in response to liver only progression. There is a growing body of evidence ^25^ suggesting that liver directed treatments such as metastasectomy, hepatic arterial embolization and percutaneous hepatic chemoperfusion can result in clinically meaningful periods of disease control; some approaches may additionally stimulate or boost adaptive immune responses through immunogenic cell death and dysregulation of local immune escape mechanisms. ^26,27^

## Conclusion

Pembrolizumab as a single agent can be used in the management of metastatic UM with an acceptable toxicity profile and provides clinically meaningful benefit in a significant proportion of patients. Prospective clinical trials are needed to characterize the magnitude of benefit and whether specific groups would be best served by alternative or combination treatments and determine the optimal modalities and sequencing. Further research on underlying immune escape mechanisms is needed to drive the rational design of future studies for this rare malignancy.
